# Amide proton transfer-weighted (APTw) CEST MRI in clinical routine for single time point diagnosis of pseudoprogression in IDH-wildtype glioblastoma

**DOI:** 10.1093/neuonc/noaf261

**Published:** 2025-11-13

**Authors:** Thomas Zeyen, Inga Krause, Andreas Decker, Florian Kroh, Sebastian Regnery, Johannes Weller, Niklas Schaefer, Mousa Zidan, Anna-Laura Potthoff, Matthias Schneider, Lea L Friker, Jochen Keupp, Christoph Katemann, Julian P Layer, Christina Schaub, Eleni Gkika, Hartmut Vatter, Torsten Pietsch, Alexander Radbruch, Ulrich Herrlinger, Daniel Paech

**Affiliations:** Department of Neurooncology, Centers for Neurology and Integrated Oncology (CIO), University Hospital Bonn, Bonn, Germany (T.Z., J.W., N.S., C.S., U.H.); Department of Radiology, Brigham and Womeńs Hospital, Harvard Medical School, Boston, Massachusetts (T.Z., I.K., F.K., D.P.); Department of Radiology, Brigham and Womeńs Hospital, Harvard Medical School, Boston, Massachusetts (T.Z., I.K., F.K., D.P.); Department of Neuroradiology, University Hospital Bonn, Bonn, Germany (I.K., M.Z., A.R., D.P.); German Center for Neurodegenerative Diseases (DZNE), Bonn, Germany (A.D.); Department of Radiology, Brigham and Womeńs Hospital, Harvard Medical School, Boston, Massachusetts (T.Z., I.K., F.K., D.P.); Department of Radiation Oncology, University Hospital Heidelberg, Heidelberg, Germany (S.R.); Department of Neurooncology, Centers for Neurology and Integrated Oncology (CIO), University Hospital Bonn, Bonn, Germany (T.Z., J.W., N.S., C.S., U.H.); Department of Vascular Neurology, Center for Neurology, University Hospital Bonn, Bonn, Germany (J.W.); Department of Neurooncology, Centers for Neurology and Integrated Oncology (CIO), University Hospital Bonn, Bonn, Germany (T.Z., J.W., N.S., C.S., U.H.); Department of Neuroradiology, University Hospital Bonn, Bonn, Germany (I.K., M.Z., A.R., D.P.); Department of Neurosurgery, University Hospital Bonn, Bonn, Germany (A.P., M.S., H.V.); Department of Neurosurgery, University Hospital Bonn, Bonn, Germany (A.P., M.S., H.V.); Institute of Neuropathology, University Hospital Bonn, Bonn, Germany (L.F., T.P.); Institute of Experimental Oncology, University Hospital Bonn, Bonn, Germany (L.F., J.L.); Philips Research, Hamburg, Germany (J.K., C.K.); Philips Research, Hamburg, Germany (J.K., C.K.); Institute of Experimental Oncology, University Hospital Bonn, Bonn, Germany (L.F., J.L.); Department of Radiation Oncology, University Hospital Bonn, Bonn, Germany (J.L., E.G.); Department of Neurooncology, Centers for Neurology and Integrated Oncology (CIO), University Hospital Bonn, Bonn, Germany (T.Z., J.W., N.S., C.S., U.H.); Department of Neurology, University Hospital Knappschaftskrankenhaus, Ruhr University Bochum, Bochum, Germany (C.S.); Department of Radiation Oncology, University Hospital Bonn, Bonn, Germany (J.L., E.G.); Department of Neurosurgery, University Hospital Bonn, Bonn, Germany (A.P., M.S., H.V.); Institute of Neuropathology, University Hospital Bonn, Bonn, Germany (L.F., T.P.); Department of Neuroradiology, University Hospital Bonn, Bonn, Germany (I.K., M.Z., A.R., D.P.); Department of Neurooncology, Centers for Neurology and Integrated Oncology (CIO), University Hospital Bonn, Bonn, Germany (T.Z., J.W., N.S., C.S., U.H.); Department of Radiology, Brigham and Womeńs Hospital, Harvard Medical School, Boston, Massachusetts (T.Z., I.K., F.K., D.P.); Department of Neuroradiology, University Hospital Bonn, Bonn, Germany (I.K., M.Z., A.R., D.P.)

**Keywords:** amide proton transfer-weighted imaging, APTw MRI, biomarkers, chemical exchange saturation transfer, CEST, DWI, glioblastoma, perfusion, pseudoprogression

## Abstract

**Background:**

Differentiating progressive disease (PD) from treatment-related effects (TRE) in glioblastoma remains challenging, particularly at single time point evaluations. TRE can occur at any disease stage, and its underlying biology is poorly understood. This study evaluates the clinical feasibility and diagnostic performance of amide proton transfer-weighted (APTw) MRI in this challenge.

**Methods:**

Following the integration of APTw MRI into the routine clinical workflow for brain tumor imaging, we screened a total of 870 scans from 626 patients. APTw signal (voxel-based measurement) was automatically quantified in gadolinium-enhanced T1w and FLAIR regions of interest using a deep learning-based approach for 3D tumor segmentations. PD and TRE were compared using unpaired *t*-tests, and diagnostic accuracy was assessed via ROC- and logistic regression analysis.

**Results:**

Among 256 MRI scans of 143 patients with glioblastoma, 65 scans showed PD (*n* = 42) or TRE (*n* = 23). The median APTw signal was higher in PD (2.23%) vs TRE (1.67%; *P* = .0001). ROC analysis showed an area under the curve (AUC) of 0.82. In patients with early PD or TRE (<6 months post-radiotherapy), the AUC increased to 0.93. Anti-angiogenic therapy decreased APTw signal (*P *< .01). Combining APTw MRI with DWI and PWI improved diagnostic accuracy (AUC = 0.90).

**Conclusions:**

APTw MRI is a non-invasive imaging tool that is feasible for clinical routine and aids in differentiation of early progression from pseudoprogression in glioblastoma. Its diagnostic accuracy decreases with application of anti-angiogenic treatment and at later follow-up time points. Highest diagnostic accuracy was found in a multimodal approach combining APTw MRI, PWI and DWI.

Key Points

- APTw MRI is clinically feasible and helps detect treatment-related effects, especially early post-radiotherapy.
- APTw MRI combined with PWI and DWI achieved highest diagnostic accuracy in detecting treatment-related effects in glioblastoma.

Importance of the study

Distinguishing TRE from PD in glioblastoma is a critical challenge, particularly at single time point evaluations. This study highlights the feasibility of APTw MRI in clinical routine and its value as a diagnostic tool for this differentiation. By exclusively analyzing glioblastoma, IDH-wildtype and thus distinguishing this research from prior studies, we observed significantly higher APTw signal in PD compared to TRE, with promising diagnostic accuracy, particularly at early time points (<6 months post-first-line radiotherapy). These findings suggest that APTw MRI could facilitate earlier and more precise treatment decisions. However, its diagnostic performance may be weakened by anti-angiogenic therapy and at later follow-up time points. The highest diagnostic accuracy was achieved using a multimodal imaging approach that combined APTw MRI, PWI and DWI. This study establishes APTw MRI as a promising candidate for future prospective studies investigating the role of advanced imaging techniques in glioblastoma assessment.

The treatment of glioblastoma remains challenging, with median survival still limited to 12-18 months.^1-3^ In addition to therapeutic resistance, the frequent occurrence of treatment-related effects (TRE) poses a major challenge.^4^ Different forms of TRE appear to differ biologically and are characterized by their appearance over the course of the disease, although definitions vary widely in the literature.^5^ Pseudoprogression typically occurs within the first three to six months following radiotherapy (RT), while radiation necrosis (RN) tends to develop more than six months, or even years post-RT.^6^ Immune responses, on the other hand, are associated with the administration of immunotherapy. However, effective treatment for symptomatic TRE, such as the vascular-endothelial-growth factor (VEGF)-inhibitor bevacizumab, is often delayed due to the difficulty in distinguishing it from PD. Current response assessment in Neuro-Oncology (RANO) 2.0 criteria require a follow-up MRI after 4-6 weeks for this assessment.^7^ Since TRE can be highly symptomatic, with early treatment being particularly crucial for cases of RN, an accurate diagnostic method for detection at a single time point would be highly beneficial.^5^^,^^8^ Although histopathological examination is regarded as diagnostic gold standard, it is (1) often not practically feasible due to the risks of another surgical intervention and (2) standardized histopathological criteria for this purpose do not exist, resulting in poor diagnostic agreement between neuropathologists.^9^ This underlines the need for reliable non-invasive tools to distinguish TRE from PD at the earliest time point possible without need for follow-up scans or surgical interventions.

Advanced imaging techniques such as diffusion-weighted imaging (DWI), perfusion-weighted imaging (PWI), amino acid positron emission tomography (PET), and MR spectroscopy can support the differentiation of TRE from PD, although their availability and use vary across institutions.^10-13^ These advanced modalities, whether applied individually or in combination, can improve diagnostic accuracy and support clinical decision-making.^6^ However, each of these imaging techniques has notable limitations regarding sensitivity and specificity (in the case of DWI), as well as issues with regional availability, particularly for MR spectroscopy and amino acid PET.^14^ A distinct limitation of PWI is that image acquisition protocols—such as dynamic susceptibility contrast (DSC)-PWI or arterial spin labeling (ASL)-PWI—and the cut-off values applied afterwards vary between institutions, often leading to inconsistencies in comparability.^15^ The recently updated RANO 2.0 criteria^7^ emphasize the growing evidence supporting the role of advanced imaging techniques in glioma assessment, though formal incorporation of these methods has yet to be implemented.

Chemical exchange saturation transfer (CEST) is an emerging MRI technique that allows non-invasive detection of low-concentrated metabolites and proteins, with a resolution comparable to that of conventional MRI.^16^ In brain tumor research, amide proton transfer-weighted (APTw) imaging has been established, with an FDA-approved MRI sequence available for this technique.^17^ APTw MRI does not require administration of contrast agent, and the scan time is short (3-5 minutes),^18^ making it a convenient, non-invasive tool that can be easily integrated into standardized brain tumor imaging protocols. Its potential in brain tumor assessment has already been demonstrated. For instance, APTw MRI has shown the ability to predict isocitrate dehydrogenase (IDH) mutation status or WHO grade, with IDH-mutant tumors and lower grade gliomas exhibiting lower APTw contrast values.^19-21^ Notably, some studies have identified the potential of APTw MRI for differentiation of PD and TRE.^22-26^ However, each of these studies included a limited number of subjects and, more importantly, most studies also included a considerable proportion of IDH-mutant gliomas. This is particularly relevant given that IDH-mutant tumors exhibit distinct APTw signal patterns compared to IDH-wildtype glioblastomas. Besides, most of the studies investigated APTw MRI combined with other techniques (PET, DWI or PWI), as well as without considering heterogeneous treatment and various progression time points. Solely Ma B et al.^22^ evaluated APTw MRI as single modality and demonstrated its sufficient potential to distinguish PD from TRE within three months after chemoradiation. In summary, preliminary data suggest APTw MRI as a potential tool for diagnosis of TRE in glioblastoma that warrants further investigation.

In this study, we analyzed a cohort consisting exclusively of IDH-wildtype glioblastoma (CNS WHO grade 4, as defined by the 2021 WHO classification^27^). Based on prior reports, we hypothesized that (1) APTw signal within the T1-w contrast-enhancing (CE) region is higher in cases of PD compared to TRE, and (2) APTw MRI could be a clinically feasible tool to support decision-making in this challenging clinical scenario.

## Material and Methods

### Patient Cohort and Demographics

A total of 870 APTw MRI scans from 626 brain tumor patients that were acquired between January 2020 and ­January 2023 in the clinical routine at the Neurooncology center Bonn were retrospectively screened in this analysis. 143 adult patients (>18 years) with a tissue-based diagnosis of glioblastoma, IDH-wildtype (CNS WHO grade 4) according to the 2021 WHO classification, were identified. In total, 256 MRI scans were available, comprising 74 pre-treatment scans, 21 baseline scans (post-RT, as per RANO 2.0 criteria^7^), and 161 follow-up scans acquired after initiation of first-line therapy with either RT or combined radiochemotherapy. Follow-up MRI scans were assessed based on RANO 2.0 criteria with modifications as detailed below. A total of 78 scans indicated stable disease or partial response, while 83 scans indicating suspected tumor progression, comprising 55 cases of PD and 28 cases of TRE. Of the follow-up scans indicating either PD or TRE, several were excluded for the following reasons:

1. **Leptomeningeal progression** (*n* = 1): Insufficient imaging comparability.
2. **Severe motion artifacts** (*n* = 1): Rendered APTw analysis uninterpretable.
3. **Predominantly non-enhancing tumor ROI** (*n* = 7): Exclusion due to the inability to define distinct, non-overlapping ROIs for solid tumor and peritumoral edema with the used AI-based auto segmentation HD-GLIO.
4. **Non-measurable disease** (*n* = 9): Lesions smaller than 10 × 10 mm (per RANO 2.0^7^) were excluded due to insufficient ROI size for reliable analysis in APTw map.

**Figure 1.**
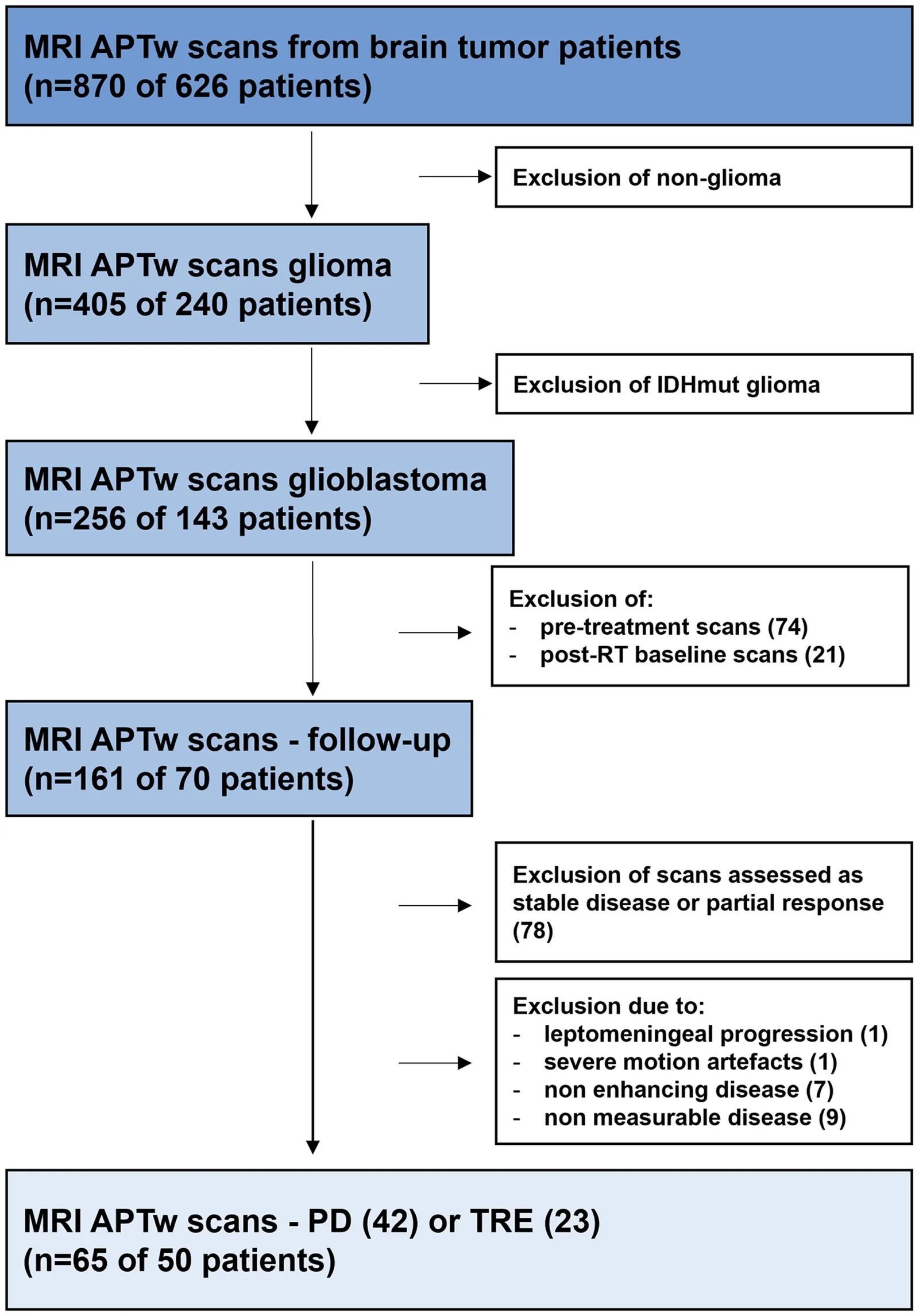
Flowchart of patient selection for analysis of MRI APTw images. A total of 870 MRI APTw scans from brain tumor patients were initially reviewed. After excluding scans from non-glioma patients and IDH-mutant glioma patients, 256 scans from 143 patients with glioblastoma were screened. The final analysis included 65 scans from 50 patients, all of whom showed either PD or TRE. PD = progressive disease; TRE = treatment-related effects.

Ultimately, 65 scans from 50 patients of suspected progression were included in the analysis, consisting of 42 scans showing unequivocal PD and 23 scans representing TRE. A detailed flowchart of scan and patient selection is provided in Figure 1.

Demographical and clinical data were collected, including age, sex, extent of resection (EOR), O6-methylguanine-DNA methyltransferase (*MGMT*) promoter methylation status, as well as information on first-line tumor therapies. Demographic data are presented in Table 1 for all patients (*n* = 50), as well as for subgroups with APTw MRI scans indicating PD (*n* = 34) or TRE (*n* = 16). Individuals were assigned to the PD or TRE group based on which event occurred first in their disease course. Supplemental table 1 provides detailed demographic information for each individual patient. Time from end of RT to suspected progression was evaluated. Additionally, survival data, including overall survival (OS) and progression-free survival (PFS), were assessed. OS was defined as the time from initial diagnosis (date of first tissue sampling) to death, while PFS was defined as the time from initial diagnosis to the first confirmed tumor progression (please see below for assessment criteria). “Time to suspected progression” was defined as the interval between initial diagnosis and the MRI demonstrating suspected progression (subsequently confirmed as either PD or TRE). Patients without recorded death or progression or with unknown outcomes were censored at the date of their last follow-up examination. The data used in this study have not been included in any previously published work.

**Table 1.** Demographic data

1.1 patient-related	All patients (*n* = 50)	PD patients (*n* = 34)	TRE patients (*n *= 16)	***P*-value** ^a^^**,**^^b^
**Age (years)**				
Median (range)	60 (31-80)	60 (31-80)	58 (38-72)	.31^a^
**Sex**				
Male (%)	31 (62)	22 (64.7)	9 (56.3)	.76^b^
**EOR**				
Biopsy (%)	6 (12)	6 (17.6)	0 (0)	
Subtotal resection (%)	20 (40)	15 (44.1)	5 (31.3)	.07^b^ (TR *vs* no TR)
Total resection (%)	24 (48)	13 (38.3)	11 (68.7)	
**MGMT methylation**				
Negative (%)	24 (48)	18 (53)	6 (37.5)	
Positive (%)	26 (52)	16 (47)	10 (62.5)	.31^b^
**First line therapy**				
RT (%)	50 (100)	34 (100)	16 (100)	>.99^b^
Systemic therapy (%)	48 (96)	32 (94.1)	16 (100)	>.99^b^
temozolomide (%)	29 (58)	22 (64.7)	7 (43.8)	.22^b^
lomustine + temozolomide (%)	17 (34)	8 (23.5)	9 (56.2)	**.03** ^b^ ^,^^*^
imatinib + temozolomide (%)	1 (2)	1 (2.9)	0 (0)	-
temsirolimus (%)	1 (2)	1 (2.9)	0 (0)	-

1.2 MRI scan related	All scans (*n* = 65)	PD scans (*n* = 42)	TRE scans (*n* = 23)	
**Time from end of RT to MRI with suspected progression**				
Median (IQR)	11.31 months (5.1-22.5)	13.5 months (6.7-22.4)	10.45 months (3.9-24.2)	.56^a^
<6 months after RT	18 (27.7)	10 (23.8)	8 (34.8)	.39^b^
>6months after RT	47 (72.3)	32 (76.2)	15 (65.2)	-
**Number of therapy lines before suspected progression**				
1 line (%)	32 (49.2)	17 (40.5)	15 (65.2)	.07^b^
2 lines (%)	27 (41.5)	22 (52.4)	5 (21.7)	^-^
>2 lines (%)	6 (9.3)	3 (7.1)	3 (13.1)	^-^

**Table 1.1** presents patient-specific demographic characteristics (*n* = 50), including age, sex, extent of resection (EOR), MGMT promoter methylation status, and details of first-line therapy. **Table 1.2** summarizes MRI-related data (*n* = 65), including the time from the end of RT to the analyzed MRI and the number of prior treatment lines at the time of the MRI. Median values are reported with corresponding ranges or interquartile ranges, while categorical variables are presented as absolute counts with percentage frequencies. *P*-values are shown in the bottom row of each table and reflect group comparisons: superscript.

Abbreviations: CNS = central nervous system; EOR = extent of resection; IDH = isocitrate dehydrogenase; IQR = interquartile range; MGMT = O6-methylguanine-DNA-methyltransferase; PD = progressive disease; RT = radiotherapy; TR = total resection; TRE = treatment-related effects; WHO = World Health Organization.

a Denotes *P*-values from the Mann–Whitney *U* test (for continuous variables), and.

b Indicates *P*-values from the Fisher’s exact test (for categorical variables).

### Progression Assessment and Identification of Treatment Related Effects

Progression assessments were performed by TZ (over four years of experience in clinical neuro-oncology and neuro-oncological imaging) and DP (over 14 years of experience in neuroimaging, with a focus on neuro-oncology imaging), based on the current RANO 2.0 criteria for high-grade gliomas in adults^7^ with modifications (see below). Each reader performed the assessment independently. Discrepancies were subsequently reviewed jointly, and a consensus decision was reached. The first post-RT MRI was considered the baseline scan. Within 12 weeks post-RT, a diagnosis of PD required confirmation via follow-up imaging or histopathology, unless new T1w-CE lesions appeared clearly outside the 80% isodose line. Non-enhancing disease was not assessed, and a > 25% increase in the maximum cross-sectional area of CE lesion was required to diagnose PD. Any new CE lesions appearing more than 12 weeks post-RT were defined as PD. At any time point, MRI evidence of new leptomeningeal dissemination was classified as PD.

TRE were defined as enlarging CE lesions that remained stable or regressed without changes in tumor therapy across at least one follow-up scan. Each follow-up MRI was compared to the preceding scan. If a lesion was later confirmed as PD, the progression date was retrospectively assigned to the earlier scan.

The following modifications to the RANO 2.0 criteria were applied:

1. In cases with >25% CE tumor increases but suspected TRE, relative cerebral blood volume (rCBV) from DSC-PWI was assessed, if available. If rCBV was not elevated, follow-up imaging after 4-8 weeks was used for final classification.
2. When bevacizumab (7.5 mg/kg, max 4 doses) was used to treat RN, lesions were classified as TRE only if they remained stable or regressed across at least three subsequent MRI scans without further bevacizumab, to avoid misclassifying pseudoresponse. Within the analyzed cohort, this scenario occurred in one patient.
3. If suspected progression led to an immediate treatment change, the scan was categorized as PD, as retrospective assessment of TRE versus treatment response was considered unreliable. In total, five patients in this cohort underwent immediate treatment change rather than early follow-up MRI.
4. Patients receiving experimental first-line therapies were included. While only two patients underwent experimental therapy, the treatment itself was assumed not to have influenced MR imaging or biomarker accuracy. Specific details are presented in Supplemental Table 1.

### MRI and APTw Imaging

MRI was performed on a Philips (Best, Netherlands) 3 T scanner (Achieva). The MRI protocol included the following sequences: 3D-fluid attenuated inversion recovery (FLAIR), 3D-T1w- before and after gadolinium injection and 3D-T2w. In alignment with the consensus recommendations on clinical APTw imaging approaches at 3 T^18^ we used the 3D APTw_asym_ provided by Philips:^28^ turbo spin echo, voxel size 1.8 × 1.8 × 6 mm^3^, field of view (FoV)= 230 × 179.7 × 60 mm^3^ (RL × AP × FH), 10 slices, TE = 8.3 ms, TR = 6.1 s, RF saturation pulse train B_1_, _rms_ = 2 μT, T_sat_ = 2 s, duty-cycle 100%, 9 frequency offsets distributed around ω = ± 3.5 ppm and one off-resonant M_0_, intrinsic B_0_ correction MTR asymmetry at + 3.5 ppm. The scan time for this APTw sequence was 3 minutes and 53 seconds.

Voxels outside the automatically generated segments were assigned a signal intensity of −10 during the online calculation of the APTw contrast values. As parenchymal structures are not expected to exhibit APTw signal values below −9.9 or above 9.9, these values were replaced with NaN during post-processing. The median APT voxel signal intensity was subsequently extracted for each segmentation. The median value was preferred as it is statistically more robust against outliers. Within the result section, the “APTw signal” refers to this median value.

### Post-Processing, Registration and Segmentation

Multimodal image processing was performed using the deep learning-based HD-GLIO^29^^,^^30^ algorithm, which registered native T1w, Gadolinium-enhanced T1w, T2w and FLAIR images and generated automated 3D segmentations of contrast-enhancing tumor region of interest (ROI, corresponding to solid tumor CE volume) and FLAIR-hyperintense peritumoral region ROI (corresponding to edema). ROIs were not overlapping in all cases and corresponding APTw images. All automatic segmentations were manually reviewed and corrected by TZ and IK (more than three years of experience in neuroimaging), using the Medical Imaging Interaction Toolkit (MITK, version v2024.06.2)). T1w-hypointense regions (resection cavities, necroses, cystic structures) and post-surgical hemorrhages were carefully excluded to prevent bias in APT quantification caused by their high protein content. Two representative cases and their respective ROIs are shown in supplemental figure 1.

### Analysis of DWI and DSC-PWI

Quantitative analysis of DWI was conducted using apparent diffusion coefficient (ADC) maps, which were available for all 65 scans. ROIs derived from contrast-enhanced T1w and FLAIR images were co-registered to the corresponding ADC maps. Voxel-wise ADC values were then extracted from these ROIs. The 10th percentile ADC value (p10) of FLAIR ROIs was selected for subsequent statistical analysis, as it demonstrated superior model performance compared to values derived from the T1w-CE ROIs.

DSC-PWI was assessed qualitatively, based on information extracted from routine clinical radiology and board reports, which were available for 62 out of 65 scans. Findings were categorized into two groups: (1) “significantly increased relative cerebral blood volume (rCBV) in the region of interest” and (2) “no significantly increased rCBV in the region of interest.”

### Statistical Analysis

Statistical analyses were performed using SPSS version 29.0.0.0 (IBM software) and Prism version 10.4.0 (GraphPad). Descriptive data analysis included the calculation of frequencies and median values for selected variables, accompanied by either the range or the interquartile range (IQR). Demographic data were compared between subgroups (PD vs TRE) using Mann-Whitney-*U*-test for continuous variables and Fisher’s exact test for categorial variables. The median value of voxel-based APTw signal for each ROI is reported with the corresponding IQR. Comparison of APTw signal values between subgroups was performed using unpaired t-test. The Shapiro-Wilk test revealed normal distribution of the APTw signal values (*P* = .57). For analysis of diagnostic accuracy receiver operating curve (ROC) analysis was performed. The event of TRE was defined as positive outcome, hereby assessing the accuracy of correctly identifying TRE. The area under the curve (AUC) is given for each graph including the 95% confidence interval (CI) and the derived p-value. The most appropriate cut-off for each ROC analysis was derived by calculating the Youden′s J (J=sensitivity+specificity-1). ROI volumes (or the number of voxels per ROI, respectively) were compared via unpaired t-test. OS and PFS were estimated according to Kaplan-Meier and reported with a median survival time and a 95% CI. Numbers at risks are provided below the Kaplan–Meier curves. Univariable and multivariable logistic regression analyses were performed to evaluate the performance of individual and combined (multimodal) approaches, including APTw MRI, DWI, and PWI.

For all statistical analyses, *P*-values <.05 were regarded as statistically significant. In the figures, significant results are marked as *=<.05, **=<.005, ***=<.0005. Since a multivariable model with predefined predictors was used, no multiple testing correction was required.

## Results

### Patient Demographics and Outcome

In the analyzed cohort of 50 glioblastoma patients with available APTw MRI at suspected progression, the median age was 60 years (range: 31-80), and 62% of the patients were male (Table 1). Total resection was achieved in around half of the patients (48%), and a MGMT promotor hypermethylation status was found in 52%. All patients received first-line RT, and 48/50 (96%) underwent additional systemic treatment, most commonly with temozolomide only (58%, *n* = 25 following Stupps protocol^31^ and *n* = 4 following Perry scheme^32^) A higher frequency of combined first-line treatment with RT and lomustine/temozolomide (following NOA-09 protocol^33^) was observed in the TRE group compared to the PD group (*P* = .03). Furthermore, TRE patients tended to have more often total resection (*P* = .07), and TRE tended to occur more commonly during or shortly after first-line therapy (*P* = .07). No patients received immunotherapy, such as immune checkpoint inhibitors, at any time point in the course of the disease. The median time from the end of RT to suspected progression was 11.3 months (IQR: 5.1-22.5). Eighteen scans (27.7%) showed suspected progression less than 6 months following RT. About half of the scans (49.2%) showed suspected progression during/after first-line treatment, while the remaining half occurred after starting at least the second treatment line (range: 2-3). If suspected progression was followed by an immediate treatment change, the corresponding scan was categorized as PD according to the modified RANO 2.0 criteria as described above. This applied to 7.7% (5/65) of all scans. From initial diagnosis and after a median follow-up of 22.5 months (IQR: 13-40), the median PFS was 9 months (95% CI: 6.1-11.8), and the median OS was 39 months (95% CI: 21.2-93.3) (Supplemental Figure 2).

### APTw Signal in Progression and Treatment Related Effects

The mean volume of T1w-CE ROIs was larger in PD (21.1 ± 14.5 ml) compared to TRE (11.1 ± 8.2 ml), though this difference did not reach statistical significance (p = 0.06). The mean volume of FLAIR ROIs was similar between groups: 78.0 ± 49.7 ml in PD vs. 79.4 ± 41.9 ml in TRE (*P* = .9; see Supplemental Table 2).

**Figure 2.**
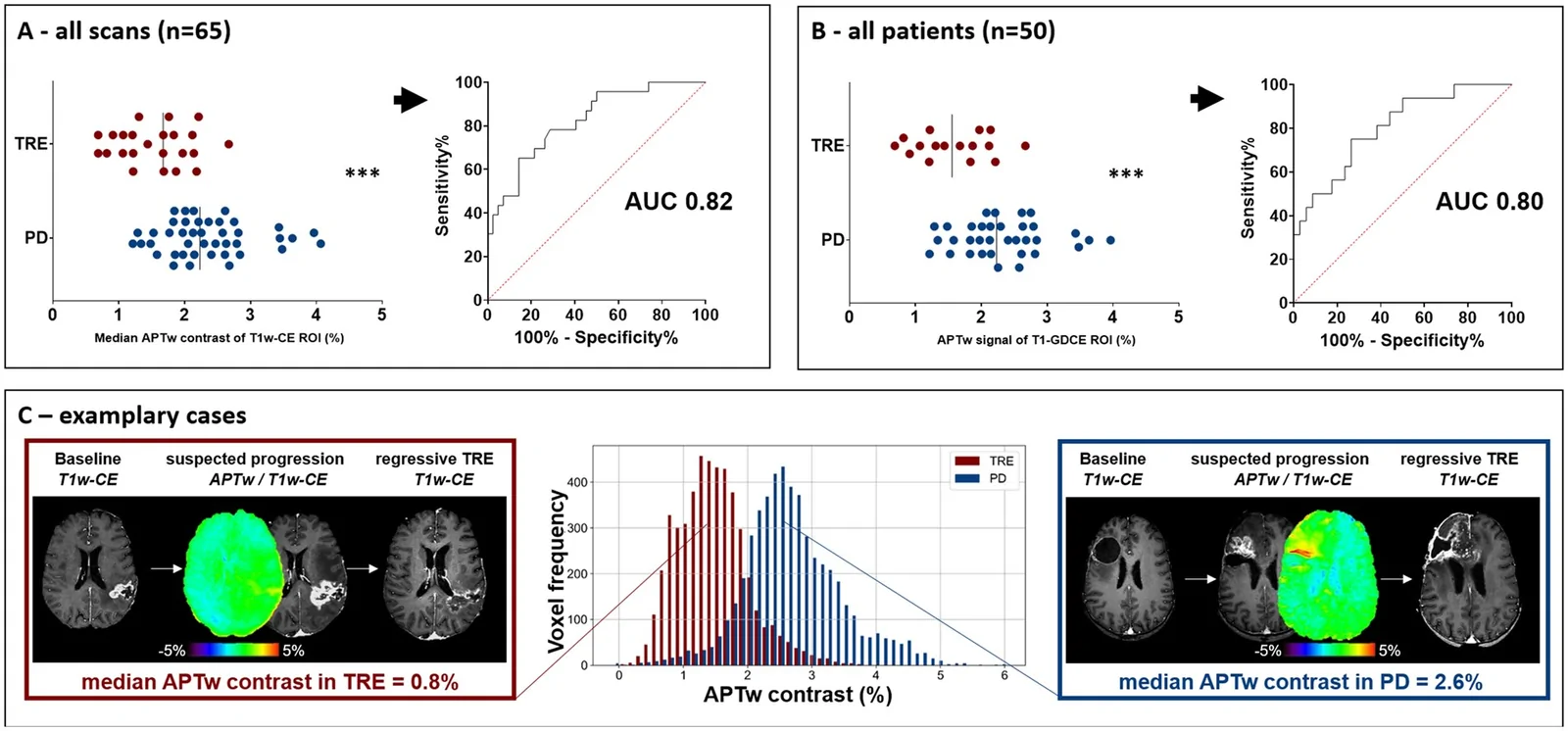
APTw signal of T1w-CE ROI in PD vs. TRE. (A) Scatter plot of median APTw contrast values (%) within T1w contrast-enhancing (T1w-CE) ROIs for all 65 scans. PD cases (blue) show significantly higher APTw signal than TRE cases (red) (Median TRE: 1.67%, IQR: 1.07-1.97; PD: 2.23%, IQR: 1.85-2.70; *P* = .0001; unpaired t-test; AUC = 0.82). (B) Same analysis restricted to one scan per patient (*n* = 50; Median TRE: 1.56%, IQR: 1.10–2.09; PD: 2.23%, IQR: 1.85-2.70; *P* = .0004; AUC = 0.80). (C) Representative APTw MRI examples from one patient with TRE (left) and one with PD (right). Imaging sequences span from baseline (last stable scan) to the time of suspected progression (including APTw MRI), and follow-up confirming PD or TRE. The PD case shows elevated APTw signal (2.6%), while the TRE case shows low signal (0.8%). Voxel-wise histograms for each ROI illustrate signal distributions. TRE = treatment-related effects; PD = progressive disease; RT = radiotherapy; ROI = region of interest.

Across all analyzed scans, the median APTw signal within T1w-CE ROIs was higher in PD (2.23%, IQR: 1.85-2.70) than in TRE (1.67%, IQR: 1.07-1.97; *P* = .0001, Figure 2A). ROC analysis demonstrated an AUC of 0.82 (95% CI: 0.72-0.93; Figure 2A), with a cut-off of 1.76% yielding a sensitivity of 65.2% and specificity of 85.7%. Within FLAIR ROIs, median APTw signal was also higher in PD (1.26%, IQR: 0.93-1.62) compared to TRE (0.75%, IQR: 0.51-1.23; *P* = .004; Supplemental Figure 3A). The corresponding AUC was 0.71 (95% CI: 0.58-0.84).

Since some patients contributed multiple APTw MRI scans, an additional analysis was performed to control for repeated measures by including only the first scan at suspected progression per patient. This analysis confirmed the primary findings: In T1w-CE ROIs, median APTw signal remained higher in PD (2.23%, IQR: 1.85-2.70) than in TRE (1.56%, IQR: 1.10-2.09; *P* = .0004; Figure 2B), with an AUC of 0.80 (95% CI: 0.67-0.93). All APTw signal values and ROC results are detailed in the supplemental table 3.

Figure 2C provides two representative cases: one TRE lesion imaged within 6 months post-RT (left), with a low APTw signal in the T1w-CE ROI of 0.8%, and one PD case (right) with a high median signal of 2.6% and increased signal on APTw images. Voxelwise histogram analysis highlights a distinct signal distribution between these two cases: while both show unimodal patterns, the TRE lesion exhibits a narrower, more homogeneous profile. In contrast, PD displays greater heterogeneity, with a broader distribution and a marked right-sided tail—indicative of high-signal voxels (∼4%). Notably, some signal overlap exists in the range of 1.8%-2.3%.

### The Time Point of Suspected Progression

**Figure 3.**
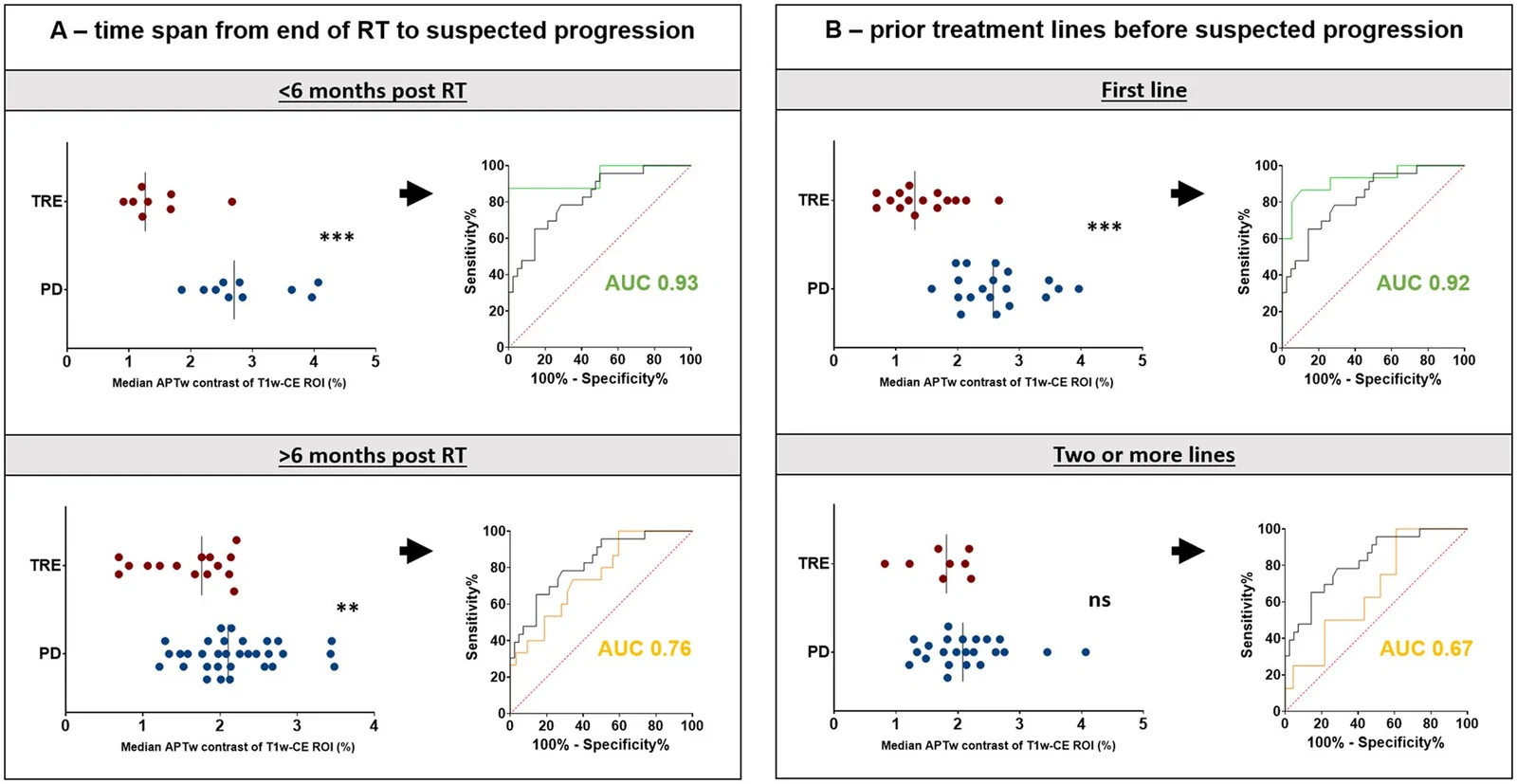
APTw signal of T1w-CE ROI in PD vs. TRE depending on time point and pretreatment. (A) Subgroup analysis based on time since end of RT. The top row shows patients imaged within 6 months post-RT, demonstrating high diagnostic accuracy (AUC = 0.93). The bottom row shows patients imaged more than 6 months post-RT, with reduced accuracy (AUC = 0.76). (B) Subgroup analysis based on pretreatment. The top row includes patients at first progression or during/after first-line therapy, showing high diagnostic performance (AUC = 0.92). The bottom row includes patients with progression following at least second-line therapy, showing lower discriminative power (AUC = 0.67).

In T1w-CE ROIs and within six months after end of RT, APTw signal was even more distinct between PD: 2.7% (2.36-3.72) and TRE: 1.26% (1.1-1.68; *P* = .0004, Figure 3A). However, more than 6 months after RT, it was less pronounced with 2.1% (1.83–2.6) for PD and 1.76% (1.06-2.12) for TRE (*P* = .002). The derived ROC curves yielded an AUC of 0.93 (0.81-1.0) for the early time point and an AUC of 0.76 (0.61-0.9) for the late (Figure 3A). In case of suspected progression less than six months post RT, the derived cut-off of 1.77% yields a sensitivity of 87.5% and a specificity of 100% for correctly identifying TRE. When considering the number of prior treatment lines, patients with suspected progression during/after first-line therapy exhibited distinct APTw signal values (PD: 2.58% [2.05-2.84] vs TRE: 1.3% [1.06-1.83], *P* < .0001, Figure 3B). However, for patients with two or more prior treatment lines, there was no significant difference (PD: 2.08 [1.77-2.47] vs TRE: 1.81 [1.34-2.17], *P* = .13). The ROC curve for patients with PD or TRE after first-line treatment showed an AUC of 0.92 with the derived cut-off of 1.99% yielding a sensitivity of 86.7% and a specificity of 89.5%.

### The Influence of Treatment on APTw MRI Findings

To evaluate potential confounding effects from varying pretreatment regimens, we performed a subgroup analysis based on the most homogeneous subgroup—patients who received first-line therapy with RT and temozolomide. In this subgroup (*n* = 28 PD vs. *n* = 7 TRE), the median APTw signal within the T1w-CE ROI was 2.42% (1.88-2.83) for PD and 1.68% (1.21-2.21) for TRE (*P* = .02), corresponding to an AUC of 0.78 (Supplemental Table 3).

**Figure 4.**
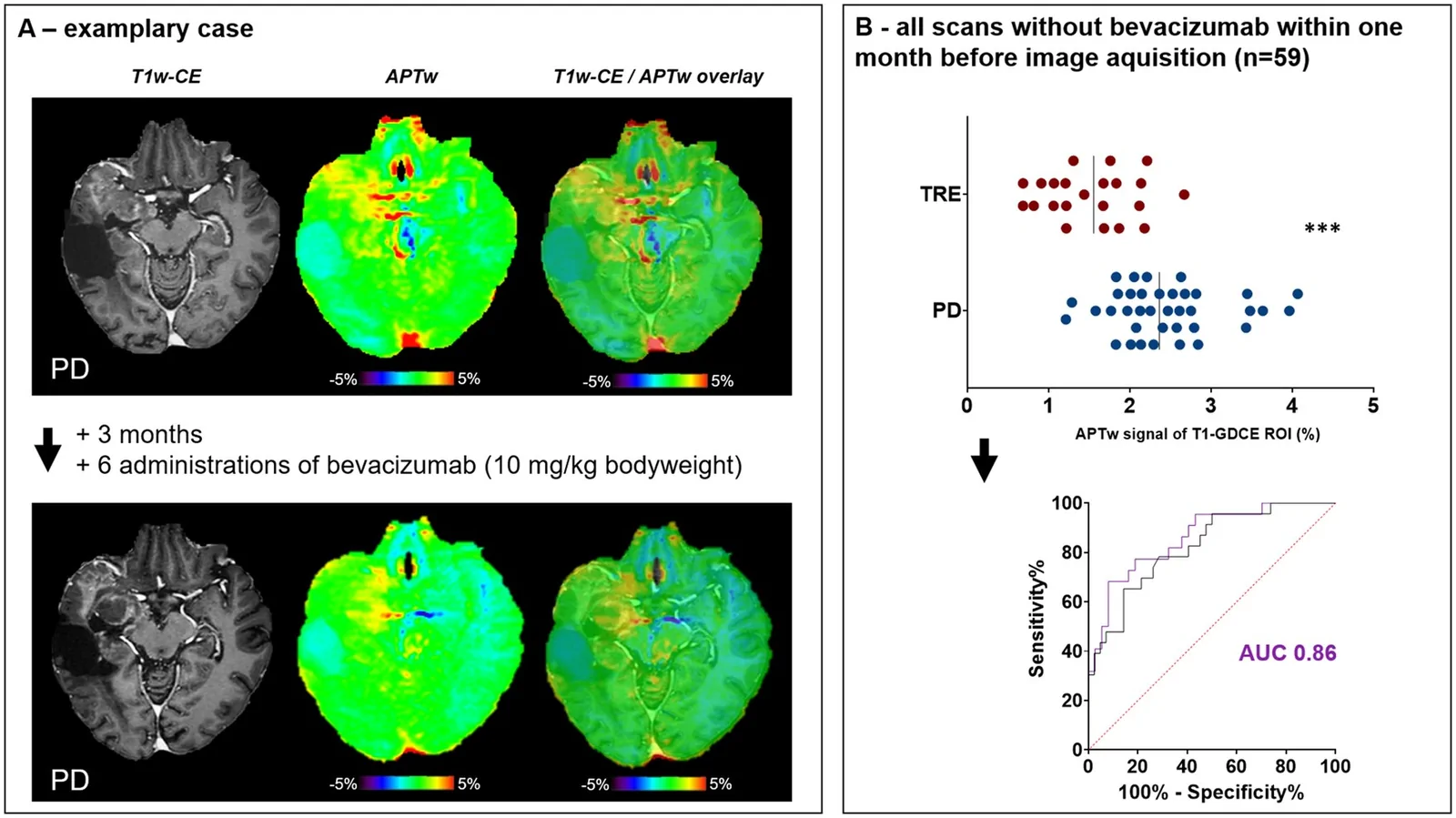
Effect of anti-angiogenic therapy on APTw MRI results. (A) Exemplary APTw MRI from one patient with PD receiving follow-up APTw MRI bevacizumab treatment (10 mg/kg bodyweight). The median APTw signal is high in the first MRI ocurrence of PD (2.4%) and decreases upon bevacizumab treatment (1.9%), although the scan demonstrates PD. (B) Scatter plot of median APTw contrast values (%) within T1w contrast-enhancing (T1w-CE) ROIs for all 59 scans after exclusion of those receiving bevacizumab within one month prior to imaging. PD cases (bottom row data points) show significantly higher APTw signal than TRE cases (top row of data points) (2.36% (2.01-2.77) in PD vs 1.56% (1.07-1.93) in TRE *P* = .0001; unpaired *t*-test; AUC = 0.86). PD = progressive disease; APTw = amide proton transfer-weighted; AUC= area under the curve.

Next, we examined whether therapies that affect vascular permeability in glioblastomas also impact diagnostic accuracy of APTw MRI. Six patients had received anti-angiogenic treatment with bevacizumab (7.5 or 10 mg/kg body weight) administered within one month prior to imaging. Supplemental table 4 provides detailed information on these patients regarding time points and outcomes. All PD (*n* = 5) or TRE (*n* = 1) occurred after substantially more than six months following RT. Of note, APTw signal was significantly lower in PD scans that were acquired after anti-angiogenic therapy (*n* = 5, 1.53%, 1.41-1.91) vs PD scans without anti-angiogenic therapy (*n* = 37, 2.36%, 2.01-2.77; *P* = .01, unpaired t-test). Figure 4A provides an example of a patient with longitudinal data available, demonstrating a decrease in APTw contrast value (from 2.4% at first PD scan without anti-angiogenic treatment to 1.9% after receiving bevacizumab 10 mg/kg bw). Comparing T1w-CE ROIs of all patients after excluding patients that received bevacizumab within one month prior to image acquisition, median APTw signal in PD was 2.36% (2.01-2.77) vs 1.56% (1.07-1.93) in TRE (*P* < .0001, Figure 4B). The derived ROC curve shows an improved AUC of 0.86 and the derived cut-off of 1.76% revealed a sensitivity of 68.2% and a specificity of 91.9% for detection of TRE.

In summary, the results suggest that the diagnostic accuracy of APTw MRI for detecting TRE decreases with increasing time since first-line RT and following the administration of anti-angiogenic therapy.

### APTw MRI Compared to DWI and DSC-PWI Analysis and Multimodal Approach

In univariable logistic regression analysis, high APTw signal and increased rCBV in PWI were significantly associated with PD (APTw MRI: AUC = 0.82, *P* < .0001; PWI: AUC = 0.76, *P* > .0001 see Table 2.1). Although low ADC values indicated PD (Odds ratio = 0.0006), model performance was not statistically significant (AUC = 0.63, *P* = .22). Notably, combining two modalities consistently improved performance over individual models, and the model incorporating all three methods achieved the highest performance, with an AUC of 0.90 (95% CI: 0.81–0.99). Detailed results of the multimodal models, including the corresponding odds ratios for each modality, are presented in Table 2.2.

**Table 2.** Predictive performance of single-modality and multimodal logistic regression models for differentiating progressive disease from treatment-related effects

*2.1 single modality performance*	AUC (95% CI)	OR (95% CI)	*P* -value
**APTw MRI** ^a^	0.82 (0.72-0.93)	10.9 (3.5-41.1)	<.0001***
**DWI** ^a^	0.63 (0.49-0.77)	0.006 (6.7e-007-19.9)	.22
**PWI** ^b^ ** [increased rCBV]**	0.76 (0.63-0.89)	10.5 (3.3-37.5)	<.0001***
***2.2 multimodal performance***			
**APTw MRI** ^a^ ** and DWI^a^**	0.84 (0.74-0.93)		
**– APTw MRI**		10.3 (3.3-44.4)	<.0001***
**– DWI**		0.009 (7.833e-008 – 769.5)	0.42
**APTw MRI** ^a^ ** and PWI** ^b^	0.89 (0.8-0.99)		
**– APTw MRI**		15.6 (3.9-104.8)	<.0001***
**– PWI [increased rCBV]**		17 (3.7-113.2)	.0001**
**DWI^a^ and PWI** ^b^	0.79 (0.67-0.91)		
**– DWI**		0.16 (5.396e-006 - 1896)	.7
**– PWI [increased rCBV]**		10.1 (3.1-36.6)	<.0001***
**APTw MRI** ^a^ ** and DWI and PWI** ^b^	0.9 (0.81-0.99)		
**– APTw MRI**		15.2 (3.8-101.7)	<.0001***
**– DWI**		0.05 (9.719e-008 - 26981)	.66
**– PWI [increased rCBV]**		16.5 (3.5-110.3)	.0002***

AUC values with 95% confidence intervals (CIs) reflect model discrimination. Odds ratios (ORs) and p-values correspond to the contribution of each modality within the model. Table 2.1 shows results of univariable logistic regression analysis and 2.2 provides results on multivariable (=multimodal) models. Multimodal combinations consistently improved performance over single modalities, with the highest AUC observed when all three methods (APTw MRI, DWI, and PWI) were combined. Superscript.

Abbreviations: APTw= amide proton transfer-weighted; AUC= area under the curve; CI= confidence interval; DWI= diffusion weighted imaging; OR= odds ratio; PWI= perfusion weighted imaging.

a Indicates continuous variables and.

b Indicates categorial variable. OR for PWI refers to “increased rCBV,” indicating that increased rCBV increases the risk for assessment as PD.

The models consistently indicate that high APTw signal within the T1w-CE ROI, elevated rCBV, and low ADC values within the FLAIR ROI are predictive of PD rather than TRE.

## Discussion

This study demonstrates the feasibility of integrating APTw MRI into clinical routine brain tumor imaging and supports its value in distinguishing PD from TRE in patients with IDH-wildtype glioblastoma (CNS WHO grade 4). We found significantly lower APTw signal in TRE—especially within the first six months after RT—possibly enabling diagnosis of TRE at a single time point. Of note, best diagnostic performance was achieved when combining APTw MRI, PWI and DWI. However, treatments affecting vascular permeability, such as anti-VEGF therapy, appear to decrease APTw signal independently of disease status, complicating interpretation.

Incorporation of APTw MRI into routine protocols proved practical. With minimal added scan time (<4 minutes^18^) and no need for additional contrast agents, it enabled the investigation of a large study sample of >800 brain tumor scans, and finally 65 glioblastoma scans with unequivocal PD (*n* = 42) or TRE (*n* = 23) were included. Focusing on IDH-wildtype glioblastoma ensured consistency, as APTw signal has been reported to be significantly lower in IDH-mutant gliomas.^19^^,^^20^ To conclude, APTw MRI showed to be a safe, time-efficient, and easily adoptable addition to standard follow-up MRI protocols at 3 Tesla for glioblastoma patients with FDA and CE approval already available for several vendors, as applied in this study.

APTw signal was significantly higher in PD than in TRE, particularly in T1w-CE tumor regions, consistent with prior research.^34^ However, diagnostic accuracy when assessing T1w-CE tumor was moderate (AUC 0.82, sensitivity 65%, specificity 85%). These findings align with previous work (eg Paprottka et al.^3^ AUC 0.85). Only Ma et al.^2^ reported higher accuracy (AUC 0.93), but this was limited to imaging within three months post RT. Our dataset al.owed for unique, longitudinal subgroup analyses of a homogeneous patient population. APTw MRI was most accurate in differentiating PD from TRE within six months of end of RT (AUC 0.93) and at first progression (AUC 0.92), with high specificity. Accuracy declined at later follow-up time points (AUC 0.76 after 6 months; AUC 0.67 at ≥2nd progression). This distinct diagnostic accuracy might arise through the heterogeneity in the TRE group, consisting of RN and pseudoprogression. Although both share similar conventional imaging features, there is evidence that they differ regarding underlying biology, histopathological features, and prognosis.^6^^,^^35^^,^^36^ This highlights the relevance of the time point within the disease course and the associated biological variability when interpreting imaging data.

Treatment heterogeneity also must be considered as a potential confounder for imaging results. In patients who received standard RT and temozolomide, APTw signals remained significantly different between PD and TRE, but diagnostic accuracy declined slightly (AUC 0.78), likely due to smaller sample sizes. Bevacizumab treatment is known to complicate progression assessment in glioma as it can lead to pseudoresponses.^37^ The results of our study suggests that bevacizumab—when administered within one month before scanning—reduced APTw signals in all patients, raising the risk of false-negative PD assessments and aligning with prior work (eg Park et al.^8^). Notably, the biological rationale of decreased APTw signal post-anti-VEGF remains elusive and seems to exceed an effect that would typically be expected from treatment response (ie decreased protein content, cellularity and cytoplasmic disruption cellularity^39^).

Although our results suggest that APTw MRI may have comparable diagnostic accuracy to PWI and DWI, multimodal models clearly outperformed single-modality approaches. This underscores the value of combining (imaging-)biomarkers to improve diagnostic accuracy. Future prospective studies should directly compare APTw MRI with other advanced imaging techniques and include additional histopathological correlation to better understand the biological underpinnings of the APTw signal.

Finally, the AI-based automated ROI segmentation used in this study provides objective and reproducible APTw measurements, but it currently requires specialized expertise and substantial post-processing time, which limits its routine use. For broader clinical adoption, a visually guided and dichotomous interpretation of APTw color maps could represent a more practical alternative. Although this strategy would likely improve feasibility in everyday workflow, its diagnostic performance remains uncertain and may be affected by inter-reader variability. Our results suggest that a predominantly homogeneous low APTw signal may favor TRE, whereas heterogeneous or markedly elevated APTw signal patterns tend to indicate PD. Future studies are needed to develop and validate standardized visual assessment criteria.

### Limitations and Outlook

Several limitations should be acknowledged. This retrospective study involved a highly selected subcohort from a large study population with an outcome that is clearly improved compared to real-world datasets (likely due to higher amount of MGMT methylated glioblastoma and total resection rate). Although most patients received temozolomide-based radiochemotherapy as first-line treatment, regimens were heterogeneous and two patients received experimental first-line therapy: one with RT + temsirolimus and another with RT + temozolomide + imatinib.

APTw MRI can be readily incorporated into standard imaging protocols. However, the focus on T1w-CE–enhancing tumors in this study excluded roughly 10% of patients with predominantly non-enhancing lesions. This represents a limitation for comprehensive clinical implementation.

Notably, ground truth accuracy might be limited in general, as current histopathological criteria cannot reliably distinguish pseudoprogression from RN,^40^ as well as specifically in this study because inter-observer variability through modified RANO 2.0 assessment might introduce bias.^41^ Although the modified RANO 2.0 criteria may enhance reference standard accuracy, the ground truth remains inherently imperfect. Additionally, classifying patients with immediate treatment changes—rather than early follow-up MRI—at suspected progression as PD may have introduced bias, affecting imaging biomarker accuracy. Additionally, it is important to note that mixed forms of PD and TRE present a major challenge in brain tumor response assessment. While their exact frequency is not well established, they are commonly observed in clinical practice and may occur more frequently following extensive pretreatment.^42^^,^^43^ This could further complicate data interpretation and contribute to the reduced diagnostic accuracy observed at later follow-up time points. However, the relatively high spatial resolution of CEST (compared to other metabolic imaging techniques such as MRS) may aid visualizing this heterogeneity.

While APTw MRI is biologically promising and clinically feasible, interpretation remains complex. The APT signals generally considered to primarily reflect protein content but is also sensitive to pH, temperature, and water content.^44^ Compared to standard anatomical sequences, current technical limitations—such as lower spatial resolution, partial volume effects, and susceptibility to subject motion and B0/B1 inhomogeneities—can reduce diagnostic confidence, particularly in small lesions. Nevertheless, improvements in APTw/CEST technology may help overcome these barriers.

## Conclusion

APTw MRI is a non-invasive technique that supports differentiation between PD and TRE in glioblastoma, especially in the early post-RT phase. Diagnostic performance can be even improved when using it within a multimodal imaging analysis, including PWI and DWI. Its integration into routine clinical imaging is feasible, though further validation and technical optimization are needed. Based on these findings, APTw MRI emerges as a promising candidate for future prospective studies focused on advanced imaging approaches for single time point assessment of treatment response.

## Supplementary Material

noaf261_Supplementary_Data

## Data Availability

The data underlying this article will be shared on reasonable request to one of the corresponding authors.
