# Corrigendum to: Amide proton transfer-weighted (APTw) CEST MRI in clinical routine for single time point diagnosis of pseudoprogression in IDH-wildtype glioblastoma

**DOI:** 10.1093/neuonc/noag176

**Published:** 2026-08-20

This is a corrigendum to: Thomas Zeyen, Inga Krause, Andreas Decker, Florian Kroh, Sebastian Regnery, Johannes Weller, Niklas Schaefer, Mousa Zidan, Anna-Laura Potthoff, Matthias Schneider, Lea L Friker, Jochen Keupp, Christoph Katemann, Julian P Layer, Christina Schaub, Eleni Gkika, Hartmut Vatter, Torsten Pietsch, Alexander Radbruch, Ulrich Herrlinger, Daniel Paech, Amide proton transfer-weighted (APTw) CEST MRI in clinical routine for single time point diagnosis of pseudoprogression in IDH-wildtype glioblastoma, *Neuro-Oncology*, Volume 28, Issue 3, March 2026, Pages 790–801, https://doi.org/10.1093/neuonc/noaf261

In the originally published version of this manuscript, a typographical error led to an inconsistency in a reported p-value.

In the abstract and Supplementary Table 3, this has been corrected to read “P = 0.0001” instead of “P = 0.001”.

Additionally, the reported median APTw signal within T1w-CE ROIs for treatment-related effects (TRE) is stated as “1.76%” within the Abstract, ‘Results’ section, Figure 2 caption, and Supplementary Table 3. This has been corrected to read “1.67%”.

## Supplementary Material

noag176_Supplementary_Data

