# Supplementary material for: Amide proton transfer-weighted (APTw) CEST MRI in clinical routine for single time point diagnosis of pseudoprogression in IDH-wildtype glioblastoma
Source: Neuro Oncol. 2025 Nov 13;28(3):790–801. doi: 10.1093/neuonc/noaf261 (PMC13070506; doi:10.1093/neuonc/noaf261)
Supplement: noaf261_Supplementary_Data [file noaf261_supplementary_data.zip › N-O-D-25-00298_supplemental figures_clean.docx]

**Amide Proton Transfer-weighted (APTw) CEST MRI for single time point diagnosis of pseudoprogression in IDH-Wildtype Glioblastoma**

- **Supplementary material - figures**

**Suppl. Figure 1**

**
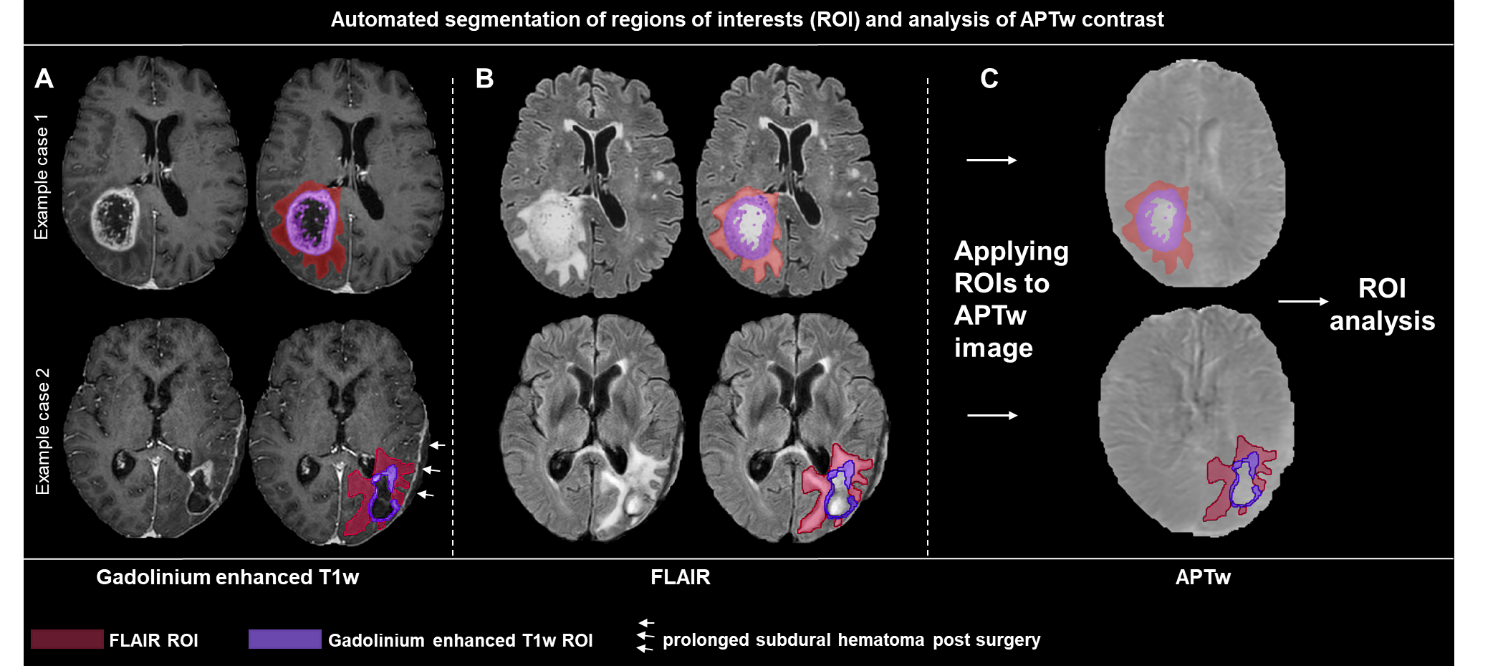
**

**Supplemental Figure 1.** Representative cases demonstrating segmentation of contrast-enhancing (CE) tumor regions (purple) and FLAIR-hyperintense peritumoral areas (red) overlaid on Gadolinium-enhanced T1-weighted (T1w) images (A) and FLAIR images (B). Necrotic, T1-hypointense regions were carefully excluded from the segmentations (see example case 1). Example case 2 illustrates the presence of concurrent, non-tumor-related contrast-enhancing structures - such as a subdural hematoma (indicated by arrows) - which were also manually excluded from the regions of interest (ROIs). Following image registration to APT-weighted (APTw) images (C), the APT signal intensity was quantified voxel wise for each ROI. Abbreviations: ROI = region of interest; FLAIR = fluid-attenuated inversion recovery.

**Suppl. Figure 2**

**
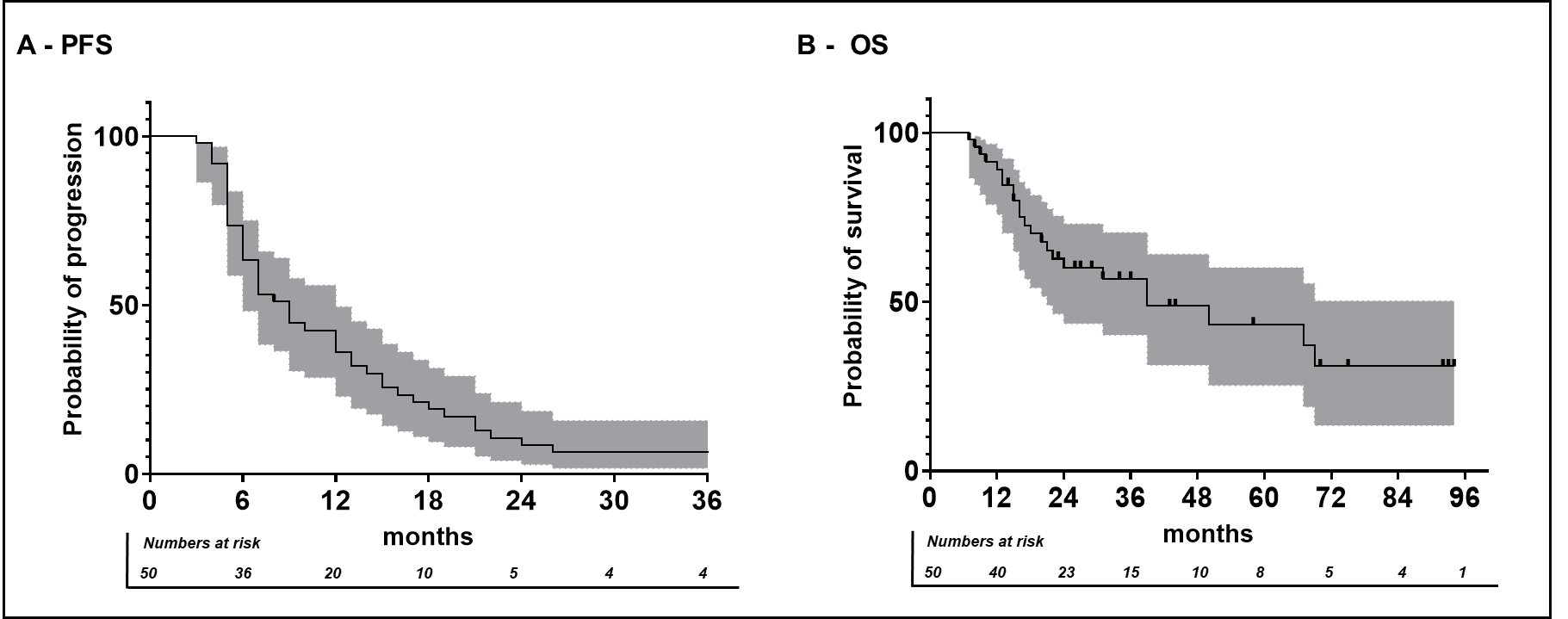
**

**Supplemental Figure 2.** Outcome in the patient cohort. Progression-free survival (PFS) (A) and overall survival (OS) (B) after the initial diagnosis of GBM are shown for all 50 patients in Kaplan–Meier curves, with ticks on the curves indicating patient censoring. Median PFS is 9 months (95% CI: 6.1–11.8), and median OS is 39 months (95% CI: 21.2–93.3). The shaded area between the dotted lines represents the 95% confidence interval (CI). Abbreviations: PFS= progression free survival; OS= overall survival.

**Suppl. Figure 3**

**
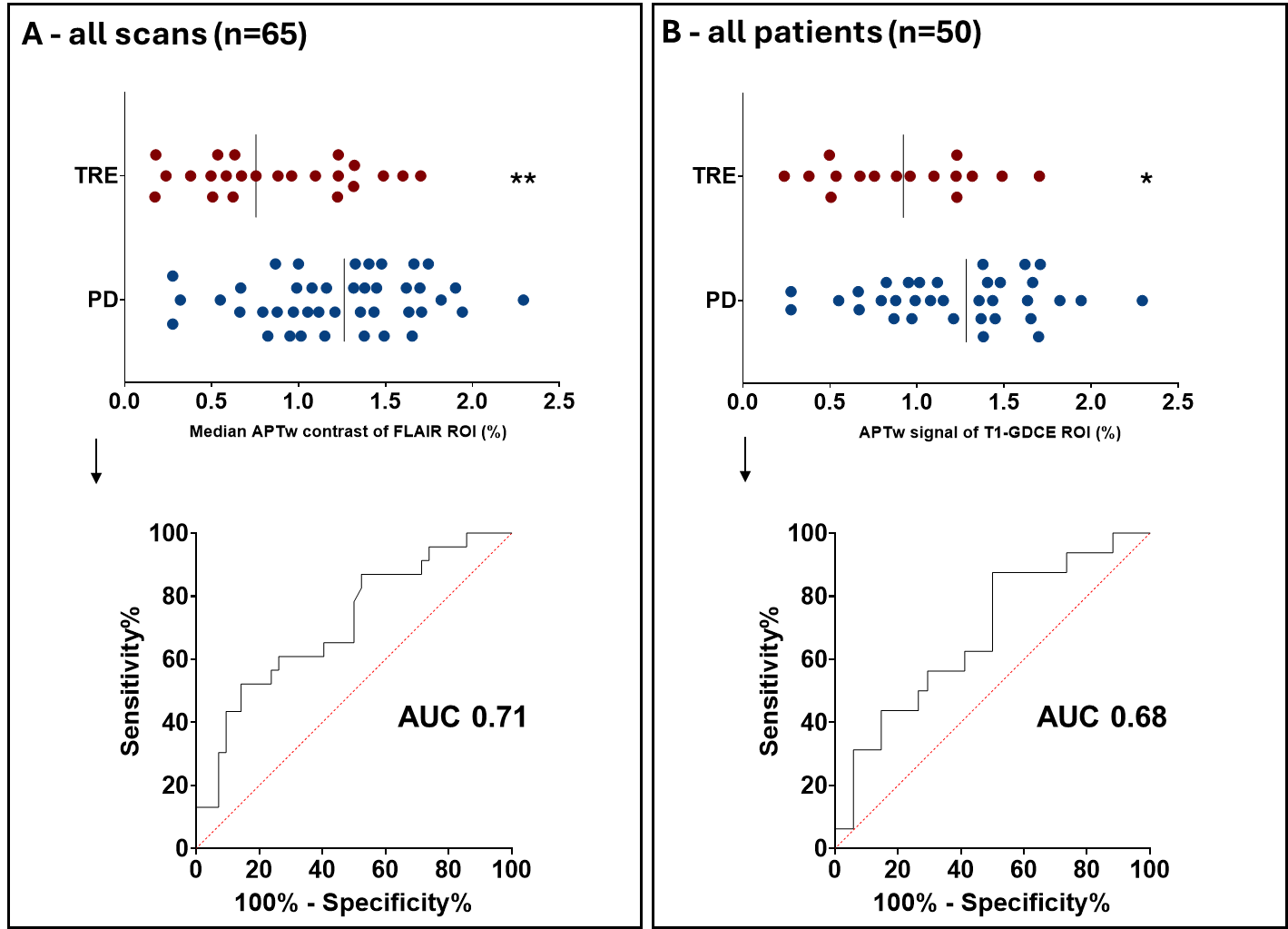
**

**Supplemental figure 3.** APTw contrast of FLAIR ROI in PD vs. TRE. (A) Scatter plot of median APTw contrast values (%) within FLAIR ROIs for all 65 scans. PD cases (blue) show significantly higher APTw contrast than TRE cases (red). (B) Same analysis restricted to one scan per patient. For exact values see supplemental table 3. Abbreviations: TRE = treatment-related effects; PD = progressive disease; RT = radiotherapy; ROI = region of interest.
