# Supplementary material for: Amide proton transfer-weighted (APTw) CEST MRI in clinical routine for single time point diagnosis of pseudoprogression in IDH-wildtype glioblastoma
Source: Neuro Oncol. 2025 Nov 13;28(3):790–801. doi: 10.1093/neuonc/noaf261 (PMC13070506; doi:10.1093/neuonc/noaf261)
Supplement: noaf261_Supplementary_Data [file noaf261_supplementary_data.zip › N-O-D-25-00298_supplemental tables_clean.docx]

**Amide Proton Transfer-weighted (APTw) CEST MRI for single time point diagnosis of pseudoprogression in IDH-Wildtype Glioblastoma**

- **Supplementary material - tables**

**Supplemental table 1 – demographic data for each individual patient**

| **patient-ID**  **(n=50)** | **age (years)** | **sex** | **MGMT** | **EOR** | **First-line therapy** | **number of APTw MRI scans (outcome)** |
| --- | --- | --- | --- | --- | --- | --- |
| apt_4 | 65 | f | unmethylated | total resection | RT + Temsirolimus | 1 (PD) |
| apt_5 | 58 | f | methylated | subtotal resection | NOA-09 protocol | 1 (PD) |
| apt_7 | 31 | m | unmethylated | biopsy | Stupp | 1 (PD) |
| apt_8 | 62 | m | methylated | subtotal resection | Stupp | 1 (PD) |
| apt_12 | 46 | m | methylated | total resection | NOA-09 protocol | 2 (all TRE) |
| apt_14 | 52 | m | unmethylated | subtotal resection | Stupp | 1 (PD) |
| apt_16 | 61 | m | unmethylated | total resection | Perry | 1 (PD) |
| apt_18 | 60 | m | unmethylated | total resection | Stupp | 1 (TRE) |
| apt_20 | 62 | f | methylated | subtotal resection | Stupp | 1 (TRE) |
| apt_21 | 72 | m | methylated | subtotal resection | Stupp | 1 (PD) |
| apt_22 | 64 | f | methylated | biopsy | NOA-09 protocol | 2 (all PD) |
| apt_24 | 52 | m | methylated | biopsy | NOA-09 protocol | 1 (PD) |
| apt_25 | 71 | m | methylated | total resection | NOA-09 protocol | 2 (all TRE) |
| apt_27 | 71 | m | unmethylated | subtotal resection | Perry | 1 (PD) |
| apt_29 | 54 | m | methylated | biopsy | NOA-09 protocol | 1 (PD) |
| apt_30 | 78 | m | methylated | biopsy | Perry | 1 (PD) |
| apt_32 | 58 | m | unmethylated | total resection | Stupp | 1 (TRE) |
| apt_33 | 57 | m | methylated | total resection | NOA-09 protocol | 3 (all TRE) |
| apt_34 | 35 | f | unmethylated | subtotal resection | Stupp | 2 (all PD) |
| apt_36 | 57 | f | methylated | total resection | NOA-09 protocol | 2 (all PD) |
| apt_41 | 62 | f | methylated | total resection | Stupp | 1 (TRE) |
| apt_44 | 60 | f | methylated | subtotal resection | NOA-09 protocol | 2 (all TRE) |
| apt_49 | 67 | f | unmethylated | subtotal resection | RT only | 1 (PD) |
| apt_56 | 53 | m | unmethylated | total resection | Stupp | 1 (PD) |
| apt_57 | 38 | f | unmethylated | total resection | Stupp | 1 (TRE) |
| apt_61 | 59 | m | unmethylated | total resection | Stupp | 3 (all PD) |
| apt_66 | 54 | m | methylated | subtotal resection | NOA-09 protocol | 1 (TRE) |
| apt_68 | 69 | m | methylated | subtotal resection | Stupp | 1 (PD) |
| apt_69 | 54 | f | methylated | subtotal resection | NOA-09 protocol | 1 (PD) |
| apt_72 | 60 | f | methylated | total resection | NOA-09 protocol | 1 (PD) |
| apt_74 | 80 | f | unmethylated | subtotal resection | RT only | 1 (PD) |
| apt_76 | 67 | m | unmethylated | subtotal resection | Stupp | 1 (PD) |
| apt_78 | 52 | m | methylated | total resection | NOA-09 protocol | 1 (PD) |
| apt_81 | 49 | f | unmethylated | subtotal resection | Stupp | 2 (1 PD; 1 TRE) |
| apt_82 | 55 | m | unmethylated | subtotal resection | Stupp | 1 (PD) |
| apt_84 | 66 | m | unmethylated | total resection | Stupp | 1 (PD) |
| apt_88 | 57 | m | unmethylated | total resection | RT + TMZ + imatinib | 3 (1 PD; 2 TRE) |
| apt_89 | 51 | f | methylated | total resection | NOA-09 protocol | 1 (TRE) |
| apt_93 | 80 | f | methylated | biopsy | Perry | 2 (all PD) |
| apt_94 | 65 | m | unmethylated | subtotal resection | Stupp | 1 (PD) |
| apt_96 | 67 | f | unmethylated | total resection | Stupp | 1 (PD) |
| apt_97 | 66 | m | methylated | total resection | NOA-09 protocol | 1 (TRE) |
| apt_104 | 55 | f | methylated | total resection | NOA-09 protocol | 1 (TRE) |
| apt_113 | 63 | f | methylated | total resection | Stupp | 2 (all PD) |
| apt_122 | 57 | m | unmethylated | subtotal resection | Stupp | 1 (PD) |
| apt_123 | 72 | m | methylated | total resection | NOA-09 protocol | 1 (TRE) |
| apt_128 | 54 | m | methylated | total resection | Stupp | 1 (PD) |
| apt_136 | 44 | m | unmethylated | total resection | Stupp | 1 (PD) |
| apt_140 | 50 | m | unmethylated | subtotal resection | Stupp | 1 (TRE) |
| apt_145 | 51 | m | methylated | subtotal resection | Stupp | 1 (PD) |

**Supplemental Table 1** – Demographic information of participants.
Supplemental Table 1 presents the demographic details of each individual included in the analysis, along with the number of APTw MRI scans per patient that were ultimately incorporated into the final dataset.

**Suppl. Table 2 – Number of voxels and volumes of ROIs**

| **N=65** | **Median number of voxels (IQR)** | **Mean volume in mm^3^ (±SD)** | **Mean volume in ml (±SD)** | **P-value** |
| --- | --- | --- | --- | --- |
| **T1w-CE ROI** - PD  -TRE | 3061 (1253 – 5178)  2056 (659 – 3903) | 21118 (±14551)  11076 (±8237) | 21.1 (±14.5)  11.1 (±8.2) | 0.06 |
| **FLAIR ROI**  - PD  -TRE | 14733 (8048 – 22241)  14143 (10388 – 21132) | 77978 (±49662)  79372 (±41910) | 78 (±49.7)  79,4 (±41.9) | 0.9 |

**Supplemental table 2 -** shows Median number of voxels (with IQR) and mean volume (with SD) of all ROIs (T1-GDCE-ROI in the upper row and FLAIR ROI in the bottom row). In the rightmost column p-value derived from unpaired t-test is given.

**Suppl. Table 3 - Median APTw contrast and derived ROC results across all subgroups**

| **1.1 CE ROIs** | **Median APT signal intensity of ROI in % (IQR)** | **P-value** | **ROC analysis – AUC (95%CI; p-value)** | **Cut-off derived from ROC (Youden Index)** | **Sensitivity (%)** | **Specificity (%)** |
| --- | --- | --- | --- | --- | --- | --- |
| **All scans (n=65)**  - PD  -TRE  **All patients (n=50)**  - PD  -TRE  **Time point (n=65)**  - PD (<6 months from RT)  - TRE (<6 months from RT)  - PD (>6 months from RT)  - TRE (>6 months from RT)  **Treatment lines (n=65)**  - PD (after first line)  - TRE (after first line)  - PD (after at least second line)  - TRE (after at least second line)  **Subgroup RT + TMZ (n=35)**  - PD  -TRE  **Excl. BEV therapy (n=59)**  - PD  - TRE | 2.23 (1.85 – 2.7)  1.76 (1.07 – 1.97)  ------------------------------  2.23 (1.85 – 2.7)  1.56 (1.10 – 2.09)  ------------------------------  2.7 (2.36 – 3.72)  1.26 (1.1 – 1.68)  2.1 (1.83 – 2.6)  1.76 (1.06 – 2.12)  ------------------------------  2.58 (2.05 – 2.84)  1.3 (1.06 – 1.83)  2.08 (1.77 – 2.47)  1.81 (1.34 – 2.17)  ------------------------------  2.42 (1.88-2.83)  1.68 (1.21-2.21)  ------------------------------  2.36 (2.01 – 2.79)  1.56 (1.07 – 1.93) | 0.001**  --------------  0.0004***  --------------  0.0004***  0.002**  --------------  <0.0001***  0.13  --------------  0.02*  --------------  <0.0001*** | 0.82 (0.72-0.93; <0.0001)  -----------------  0.80 (0.67-0.93; 0.0006)  -----------------  0.93 (0.81-1.0; 0.001)  0.76 (0.61-0.9; 0.005)  -----------------  0.92 (0.83-1.0; <0.0001)  0.67 (0.46-0.87; 0.16)  -----------------  0.78 (0.59-0.97; 0.02)  -----------------  0.86 (0.76-0.96; <0.0001) | 1.76  ---------------  1.97  ---------------  1.77  2.23  ---------------  1.99  2.23  ---------------  1.84  ---------------  1.76 | 65.2  ---------------  75  ---------------  87.5  100  ---------------  86.7  100  ---------------  71.4  ---------------  68.2 | 85.7  ---------------  73.5  ---------------  100  40.6  ---------------  89.5  39.1  ---------------  78.6  ---------------  91.9 |
| **1.2 FLAIR ROIs** | **Median APT signal intensity of ROI in % (IQR)** | **P-value** | **ROC analysis – AUC (95%CI; p-value)** | **Cut-off derived from ROC (Youden Index)** | **Sensitivity (%)** | **Specificity (%)** |
| **All scans (n=65)**  - PD  -TRE  **All patients (n=50)**  - PD  -TRE  **Time point (n=65)**  - PD (<6 months from RT)  - TRE (<6 months from RT)  - PD (>6 months from RT)  - TRE (>6 months from RT)  **Treatment lines (n=65)**  - PD (after first line)  - TRE (after first line)  - PD (after at least second line)  - TRE (after at least second line  **Subgroup RT + TMZ (n=35)**  - PD  -TRE  **Excl. BEV therapy (n=59)**  - PD  - TRE | 1.26 (0.93 – 1.62)  0.75 (0.51 – 1.23)  ------------------------------  1.28 (0.87-1.62)  0.92 (0.51-1.23)  ------------------------------  1.43 (1.06 – 1.76)  0.58 (0.3 – 1.14)  1.16 (0.84 – 1.48)  0.96 (0.58 – 1.32)  ------------------------------  1.37 (0.99 – 1.7)  0.67 (0.51 – 1.22)  1.16 (0.88 – 1.45)  1.04 (0.53 – 1.45)  ------------------------------  1.35 (0.87-1.66)  0.67 (0.50-1.22)  ------------------------------  1.36 (0.99 – 1.64)  0.71 (0.5 – 1.23) | 0.004**  --------------  0.04*  --------------  0.0007***  0.19  --------------  0.007**  0.29  --------------  0.07  --------------  0.0003*** | 0.71 (0.58-0.84; 0.005)  -----------------  0.68 (0.55-0.84; 0.04)  -----------------  0.91 (0.78-1.0; 0.003)  0.62 (0.44-0.79; 0.19)  -----------------  0.76 (0.6-0.93; 0.009)  0.62 (0.37-0.87; 0.33)  -----------------  0.73 (0.53-0.94; 0.06)  -----------------  0.77 (0.65-0.89; 0.0006) | 0.77  ---------------  1.36  ---------------  1.3  0.77  ---------------  1.29  0.77  ---------------  0.73  ---------------  1.32 | 52.2  ---------------  87.5  ---------------  100  46.7  ---------------  93.3  50  ---------------  57.1  ---------------  90.9 | 85.7  ---------------  47.1  ---------------  70  81.3  ---------------  57.9  86.9  ---------------  85.7  ---------------  54 |

**Supplemental Table 3.** Median APTw contrast and derived ROC results across all subgroups. This table presents the median APTw contrasts for all subgroups, as detailed in the manuscript, with values reported for T1w-CE ROI (upper row) and FLAIR-ROI (bottom row). The median values are accompanied by their interquartile ranges (IQRs). The third column from the left displays the p-values obtained from unpaired t-tests. The middle column provides the area under the curve (AUC) from ROC analysis for each group comparison, including the 95% confidence intervals (95% CI) and associated p-values. The third row from the right contains the ROC-derived cut-off values determined using the Youden index. The two rightmost columns report the sensitivity and specificity corresponding to these cut-off values**.**

**Supplemental table 4 – patients receiving anti-angiogenic treatment with bevacizumab**

| **Patient ID** | **Anti-angiogenic treatment (dosage) within one month before scan** | **Time span between end of RT and MRI scan** | **Outcome** |
| --- | --- | --- | --- |
| apt_24 | Bevacizumab (7.5 mg/kg bw) | 13.6 months | Progressive disease |
| apt_30 | Bevacizumab (10 mg/kg bw) | 9.9 months | Progressive disease |
| apt_56 | Bevacizumab (10 mg/kg bw) | 25.6 months | Progressive disease |
| apt_61 | Bevacizumab (10 mg/kg bw) | 31.9 months | Progressive disease |
| apt_84 | Bevacizumab (10 mg/kg bw) | 10.8 months | Progressive disease |
| apt_123 | Bevacizumab (7.5mg/kg bw) | 10.6 months | Treatment related effects |

**Supplemental table 4.** Detailed information on patients that were excluded for the subgroup analysis of patients that did not receive anti-angiogenic treatment within one month before APTw MRI scan.
