# Supplementary material for: Corrigendum to: Amide proton transfer-weighted (APTw) CEST MRI in clinical routine for single time point diagnosis of pseudoprogression in IDH-wildtype glioblastoma
Source: Neuro Oncol. 2026 Aug 20;28(10):e34. doi: 10.1093/neuonc/noag176 (PMC13635589; doi:10.1093/neuonc/noag176)
Supplement: Supplementary file 1 [file noag176_supplementary_data.pdf]

Amide Proton Transfer-weighted (APTw) CEST MRI for single time point diagnosis of pseudoprogression in IDH-Wildtype Glioblastoma

- **Supplementary material - tables**

Supplemental table 1 – demographic data for each individual patient

patient-ID (n=50)	age (years)	sex	MGMT	EOR	First-line therapy	number of APTw MRI scans (outcome)
apt_4	65	f	unmethylated	total resection	RT + Temsirolimus	1 (PD)
apt_5	58	f	methylated	subtotal resection	NOA-09 protocol	1 (PD)
apt_7	31	m	unmethylated	biopsy	Stupp	1 (PD)
apt_8	62	m	methylated	subtotal resection	Stupp	1 (PD)
apt_12	46	m	methylated	total resection	NOA-09 protocol	2 (all TRE)
apt_14	52	m	unmethylated	subtotal resection	Stupp	1 (PD)
apt_16	61	m	unmethylated	total resection	Perry	1 (PD)
apt_18	60	m	unmethylated	total resection	Stupp	1 (TRE)
apt_20	62	f	methylated	subtotal resection	Stupp	1 (TRE)
apt_21	72	m	methylated	subtotal resection	Stupp	1 (PD)
apt_22	64	f	methylated	biopsy	NOA-09 protocol	2 (all PD)
apt_24	52	m	methylated	biopsy	NOA-09 protocol	1 (PD)
apt_25	71	m	methylated	total resection	NOA-09 protocol	2 (all TRE)
apt_27	71	m	unmethylated	subtotal resection	Perry	1 (PD)
apt_29	54	m	methylated	biopsy	NOA-09 protocol	1 (PD)
apt_30	78	m	methylated	biopsy	Perry	1 (PD)
apt_32	58	m	unmethylated	total resection	Stupp	1 (TRE)
apt_33	57	m	methylated	total resection	NOA-09 protocol	3 (all TRE)

apt_34	35	f	unmethylated	subtotal resection	Stupp	2 (all PD)
apt_36	57	f	methylated	total resection	NOA-09 protocol	2 (all PD)
apt_41	62	f	methylated	total resection	Stupp	1 (TRE)
apt_44	60	f	methylated	subtotal resection	NOA-09 protocol	2 (all TRE)
apt_49	67	f	unmethylated	subtotal resection	RT only	1 (PD)
apt_56	53	m	unmethylated	total resection	Stupp	1 (PD)
apt_57	38	f	unmethylated	total resection	Stupp	1 (TRE)
apt_61	59	m	unmethylated	total resection	Stupp	3 (all PD)
apt_66	54	m	methylated	subtotal resection	NOA-09 protocol	1 (TRE)
apt_68	69	m	methylated	subtotal resection	Stupp	1 (PD)
apt_69	54	f	methylated	subtotal resection	NOA-09 protocol	1 (PD)
apt_72	60	f	methylated	total resection	NOA-09 protocol	1 (PD)
apt_74	80	f	unmethylated	subtotal resection	RT only	1 (PD)
apt_76	67	m	unmethylated	subtotal resection	Stupp	1 (PD)
apt_78	52	m	methylated	total resection	NOA-09 protocol	1 (PD)
apt_81	49	f	unmethylated	subtotal resection	Stupp	2 (1 PD; 1 TRE)
apt_82	55	m	unmethylated	subtotal resection	Stupp	1 (PD)
apt_84	66	m	unmethylated	total resection	Stupp	1 (PD)
apt_88	57	m	unmethylated	total resection	RT + TMZ + imatinib	3 (1 PD; 2 TRE)
apt_89	51	f	methylated	total resection	NOA-09 protocol	1 (TRE)
apt_93	80	f	methylated	biopsy	Perry	2 (all PD)
apt_94	65	m	unmethylated	subtotal resection	Stupp	1 (PD)
apt_96	67	f	unmethylated	total resection	Stupp	1 (PD)

apt_97	66	m	methylated	total resection	NOA-09 protocol	1 (TRE)
apt_104	55	f	methylated	total resection	NOA-09 protocol	1 (TRE)
apt_113	63	f	methylated	total resection	Stupp	2 (all PD)
apt_122	57	m	unmethylated	subtotal resection	Stupp	1 (PD)
apt_123	72	m	methylated	total resection	NOA-09 protocol	1 (TRE)
apt_128	54	m	methylated	total resection	Stupp	1 (PD)
apt_136	44	m	unmethylated	total resection	Stupp	1 (PD)
apt_140	50	m	unmethylated	subtotal resection	Stupp	1 (TRE)
apt_145	51	m	methylated	subtotal resection	Stupp	1 (PD)

Supplemental Table 1 – Demographic information of participants. Supplemental Table 1 presents the demographic details of each individual included in the analysis, along with the number of APTw MRI scans per patient that were ultimately incorporated into the final dataset.

Suppl. Table 2 – Number of voxels and volumes of ROIs

N=65	Median number of voxels (IQR)	Mean volume in mm^3 (±SD)	Mean volume in ml (±SD)	P-value
**T1w-CE ROI**<br>- PD<br>-TRE	 3061 (1253 – 5178)<br>2056 (659 – 3903)	 21118 (±14551)<br>11076 (±8237)	 21.1 (±14.5)<br>11.1 (±8.2)	 <br>0.06
**FLAIR ROI**<br>- PD<br>-TRE	 14733 (8048 – 22241)<br>14143 (10388 – 21132)	 77978 (±49662)<br>79372 (±41910)	 78 (±49.7)<br>79,4 (±41.9)	 <br>0.9

Supplemental table 2 - shows Median number of voxels (with IQR) and mean volume (with SD) of all ROIs (T1-GDCE-ROI in the upper row and FLAIR ROI in the bottom row). In the rightmost column p-value derived from unpaired t-test is given.

Suppl. Table 3 - Median APTw contrast and derived ROC results across all subgroups

1.1 CE ROIs	Median APT signal intensity of ROI in % (IQR)	P-value	ROC analysis – AUC (95%CI; p-value)	Cut-off derived from ROC (Youden Index)	Sensitivity (%)	Specificity (%)
**All scans (n=65)**						
- PD	2.23 (1.85 – 2.7)		0.82 (0.72-0.93;			
-TRE	1.67 (1.07 – 1.97)	0.0001**	<0.0001)	1.76	65.2	85.7
**All patients (n=50)**			0.80 (0.67-0.93;			
- PD	2.23 (1.85 – 2.7)					
-TRE	1.56 (1.10 – 2.09)	0.0004***	0.0006)	1.97	75	73.5
**Time point (n=65)**						
- PD (<6 months from RT)	2.7 (2.36 – 3.72)		0.93 (0.81-1.0; 0.001)			
- TRE (<6 months from RT)	1.26 (1.1 – 1.68)	0.0004***		1.77	87.5	100
- PD (>6 months from RT)	2.1 (1.83 – 2.6)		0.76 (0.61-0.9; 0.005)			
- TRE (>6 months from RT)	1.76 (1.06 – 2.12)	0.002**		2.23	100	40.6
**Treatment lines (n=65)**						
- PD (after first line)	2.58 (2.05 – 2.84)		0.92 (0.83-1.0; <0.0001)			
- TRE (after first line)	1.3 (1.06 – 1.83)	<0.0001***		1.99	86.7	89.5
- PD (after at least second line)	2.08 (1.77 – 2.47)		0.67 (0.46-0.87; 0.16)			
- TRE (after at least second line)	1.81 (1.34 – 2.17)	0.13		2.23	100	39.1
**Subgroup RT + TMZ (n=35)**						
- PD	2.42 (1.88-2.83)		0.78 (0.59-0.97; 0.02)			
-TRE	1.68 (1.21-2.21)	0.02*		1.84	71.4	78.6
**Excl. BEV therapy (n=59)**			0.86 (0.76-0.96;			
- PD	2.36 (2.01 – 2.79)					
- TRE	1.56 (1.07 – 1.93)	<0.0001***	<0.0001)	1.76	68.2	91.9
**1.2 FLAIR ROIs**	**Median APT signal intensity of ROI in % (IQR)**	**P-value**	**ROC analysis – AUC (95%CI; p-value)**	**Cut-off derived from ROC (Youden Index)**	**Sensitivity (%)**	**Specificity (%)**

**All scans (n=65)**						
- PD	1.26 (0.93 – 1.62)		0.71 (0.58-			
-TRE	0.75 (0.51 – 1.23)	0.004**	0.84; 0.005)	0.77	52.2	85.7
**All patients (n=50)**						
- PD	1.28 (0.87-1.62)		0.68 (0.55-		87.5	47.1
-TRE	0.92 (0.51-1.23)	0.04*	0.84; 0.04)	1.36		
**Time point (n=65)**						
- PD (<6 months from RT)	1.43 (1.06 – 1.76)		0.91 (0.78-			
- TRE (<6 months from RT)	0.58 (0.3 – 1.14)	0.0007***	1.0; 0.003)	1.3	100	70
- PD (>6 months from RT)	1.16 (0.84 – 1.48)		0.62 (0.44-			
- TRE (>6 months from RT)	0.96 (0.58 – 1.32)	0.19	0.79; 0.19)	0.77	46.7	81.3
**Treatment lines (n=65)**						
- PD (after first line)	1.37 (0.99 – 1.7)		0.76 (0.6- 0.93; 0.009)			
- TRE (after first line)	0.67 (0.51 – 1.22)	0.007**		1.29	93.3	57.9
- PD (after at least second line)	1.16 (0.88 – 1.45)		0.62 (0.37-			
- TRE (after at least second line	1.04 (0.53 – 1.45)	0.29	0.87; 0.33)	0.77	50	86.9
**Subgroup RT + TMZ (n=35)**						
- PD	1.35 (0.87-1.66)		0.73 (0.53-			
-TRE	0.67 (0.50-1.22)	0.07	0.94; 0.06)	0.73	57.1	85.7
**Excl. BEV therapy (n=59)**			0.77 (0.65-			
- PD	1.36 (0.99 – 1.64)		0.89;			
- TRE	0.71 (0.5 – 1.23)	0.0003***	0.0006)	1.32	90.9	54

Supplemental Table 3. Median APTw contrast and derived ROC results across all subgroups. This table presents the median APTw contrasts for all subgroups, as detailed in the manuscript, with values reported for T1w-CE ROI (upper row) and FLAIR-ROI (bottom row). The median values are accompanied by their interquartile ranges (IQRs). The third column from the left displays the p-values obtained from unpaired t-tests. The middle column provides the area under the curve (AUC) from ROC analysis for each group comparison, including the 95% confidence intervals (95% CI) and associated p-values. The third row from the right contains the ROC-derived cut-off values determined

using the Youden index. The two rightmost columns report the sensitivity and specificity corresponding to these cut-off values.

Supplemental table 4 – patients receiving anti-angiogenic treatment with bevacizumab

Patient ID	Anti-angiogenic treatment (dosage) within one month before scan	Time span between end of RT and MRI scan	Outcome
apt_24	Bevacizumab (7.5 mg/kg bw)	13.6 months	Progressive disease
apt_30	Bevacizumab (10 mg/kg bw)	9.9 months	Progressive disease
apt_56	Bevacizumab (10 mg/kg bw)	25.6 months	Progressive disease
apt_61	Bevacizumab (10 mg/kg bw)	31.9 months	Progressive disease
apt_84	Bevacizumab (10 mg/kg bw)	10.8 months	Progressive disease
apt_123	Bevacizumab (7.5mg/kg bw)	10.6 months	Treatment related effects

Supplemental table 4. Detailed information on patients that were excluded for the subgroup analysis of patients that did not receive anti-angiogenic treatment within one month before APTw MRI scan.
